# Poor early cortical differentiation of speech predicts perceptual difficulties of severely hearing-impaired listeners in multi-talker environments

**DOI:** 10.1038/s41598-020-63103-7

**Published:** 2020-04-09

**Authors:** Brandon T. Paul, Mila Uzelac, Emmanuel Chan, Andrew Dimitrijevic

**Affiliations:** 1 Evaluative Clinical Sciences Platform, Sunnybrook Research Institute, Toronto, ON M4N 3M5 Canada; 2 Otolaryngology-Head and Neck Surgery, Sunnybrook Health Sciences Centre, Toronto, ON M4N 3M5 Canada; 3 Faculty of Medicine, Otolaryngology-Head and Neck Surgery, University of Toronto, Toronto, ON M5S 1A1 Canada

**Keywords:** Sensory processing, Auditory system, Cortex, Cognitive neuroscience, Attention

## Abstract

Hearing impairment disrupts processes of selective attention that help listeners attend to one sound source over competing sounds in the environment. Hearing prostheses (hearing aids and cochlear implants, CIs), do not fully remedy these issues. In normal hearing, mechanisms of selective attention arise through the facilitation and suppression of neural activity that represents sound sources. However, it is unclear how hearing impairment affects these neural processes, which is key to understanding why listening difficulty remains. Here, severely-impaired listeners treated with a CI, and age-matched normal-hearing controls, attended to one of two identical but spatially separated talkers while multichannel EEG was recorded. Whereas neural representations of attended and ignored speech were differentiated at early (~ 150 ms) cortical processing stages in controls, differentiation of talker representations only occurred later (~250 ms) in CI users. CI users, but not controls, also showed evidence for spatial suppression of the ignored talker through lateralized alpha (7–14 Hz) oscillations. However, CI users’ perceptual performance was only predicted by early-stage talker differentiation. We conclude that multi-talker listening difficulty remains for impaired listeners due to deficits in early-stage separation of cortical speech representations, despite neural evidence that they use spatial information to guide selective attention.

## Introduction

In multi-talker settings, listeners can direct their focus of attention to one talker despite the presence of nearby competing talkers^1,2^. This attentional selection is in part dependent upon external cues, such as pitch, timing, and spatial differences between talkers’ voices, which help listeners form separate internal representations (or “objects”) for each talker^3^ so that the chosen speech signal is tracked and understand.

Neural mechanisms that support selective attention may either enhance the internal representation of an attended talker, or suppress representations of unwanted talkers. For instance, it is well established that cortical responses known to entrain to temporal fluctuations of the speech envelope (i.e., “cortical speech tracking”^4^), are larger for an attended talker over unattended talkers^5–13^. In addition, listeners can leverage spatial information to assist selective attention. In two-talker settings, an increase in oscillatory alpha (7–14 Hz) power lateralized to the hemisphere ipsilateral to the attended talker (thus representing the ignored speech)^14–17^ is suggested to reflect the synchronization of inhibitory neural circuits in multiple cortical sources that may suppress unwanted signals and direct the focus of attention in space^18,19^.

Hearing impairment, however, is well known to significantly disrupt selective attention in multi-talker environments^20,21^. Attention challenges remain despite the assistance of hearing aids^22^ or cochlear implants (CIs)^23^, suggesting that rehabilitation is incomplete or that prostheses do not adequately address the challenges that listeners face in complex auditory environments. Neural correlates of selective attention are similarly affected; enhanced cortical tracking of attended talkers is reduced in both hearing-aided^24^ and unaided impaired listeners^25^, and unaided hearing-impaired listeners show weaker lateralization of alpha power in spatial attention tasks^26^.

Despite these differences, impaired listeners appear to perform selective attention tasks above chance (e.g.,^25^), suggesting that neural correlates of selective attention to some degree are still in operation. Here we sought to discover what these neural correlates are, and which are important determinants of perception. Answers to these unresolved problems can at once shed light on properties of neural dynamics that support listening in complex environments, and explain why selective attention challenges remain in hearing-impaired individuals despite the use of hearing prostheses.

In the current study, normal-hearing and hearing-impaired adults were asked to attend to one of two identical talkers in a diotic listening task while cortical activity was recorded using electroencephalography (EEG). In particular, we focused on individuals with severe hearing impairment who have been treated bilaterally with CIs. In brief, we observed the well-known enhancement of speech tracking for attended talkers in normal-hearing listeners at early cortical stages (~150 ms), but this effect was absent in CI users at a group level. However, differentiation of attended and ignored speech was stronger for CI users at a later cortical stage (~250 ms), and favored a stronger representation of the ignored talker. CI users also showed stronger spatial attention through alpha lateralization, which was absent at a group level in normal-hearing controls. Despite these findings, CI users’ behavioral ability to report the spoken content of the attended talker was not predicted by late cortical differentiation of speech or spatial suppression of the ignored talker, but only by the extent to which the attended talker was enhanced during early cortical processing.

## Materials and methods

### Participants

Seventeen adults fitted with left and right (i.e., bilateral) CIs were recruited through Sunnybrook Health Sciences Centre Department of Otolaryngology. One CI user had a technical failure during EEG recording and was excluded from the analysis. The final sample included ten males and six females who were aged from 18 to 69 (mean age = 49.4, SD = 16.7). Participants were required to have used left and right devices together for at least one year following initial activation of their most recent implant. CI users used their left-sided devices from 3.3 to 18 years (M = 8.4 years, SD = 4.9) and their right sided devices from 3.1 to 23.3 years (M = 9.6 years, SD = 6.4). The range of delay between implantation was between zero and 16 years (M = 5.3 years, SD = 4.7).

In addition, 14 age-matched participants (age range 19 to 74, mean age = 51.3, SD = 18.2; no significant difference from CI users; *t*(28) = 0.3, *p* = 0.77), including five males and nine females, were recruited to serve as a control group. Hearing ability was measured using pure-tone audiometry in octave steps from 250 to 8000 Hz for each ear separately. All control participants had age-appropriate hearing thresholds, with nine participants aged under 68 years producing thresholds less than 25 dB hearing level (HL) up to 8 kHz in both ears, and five participants above this age had thresholds to 35 dB HL at 4 kHz, and to 55 dB HL at 8 kHz.

Herein, bilateral CI users are referred to as the BiCI group, and the normal-hearing participants will comprise the NH group. All participants were informed verbally and in writing of all experimental procedures, and provided written consent. All experimental protocols used in this study were approved by the Research Ethics Board (REB) at Sunnybrook Health Sciences Centre (#474–2016). All the methods used in this study were performed in accordance to the guidelines and regulations outlined by the Research Ethics Board (REB) at Sunnybrook Health Sciences Centre (#474–2016). The approved protocol was in agreement with the Declaration of Helsinki. Participants were compensated with money for their participation, and were provided full reimbursement for parking at the hospital campus.

### Stimuli, material, and testing environment

Broadly, the experimental task presented two concurrent series (or “streams”) of spoken digits (numbers) originating from two free-field speakers. Participants were required to attend to one speaker or the other, and then report the last digit that was perceived on the attended side. Participants were seated at the center of a circular ring array of eight speakers, positioned at 0, +/− 45, +/− 90, +/− 135, and 180 degrees relative to the listener. Digit stimuli originated only from the + /− 45-degree speakers. The cone of the speakers was set to a height of 100 cm from the ground, and each speaker cone was 80 cm away from the center. The participant, speaker array, and a computer monitor interface were contained within a sound-attenuated and electrically shielded booth.

All individual stimuli (either from the left or right speaker) were monosyllabic digits spoken by the same female talker of standard American English. Spoken digits were the numbers 0 through 9 excluding the disyllabic number 7. “0” was pronounced as “Oh” (/oω/). The duration of each digit lasted from 434 to 672 milliseconds (ms), and to equate duration, the length of the sound file was appended with zeros to reach 695 ms. The root mean squared (RMS) amplitude of all digits was adjusted by a scale factor so that they were presented at equal intensity. The digits were presented at 70 dBA as measured at the center of the ring array. During the time the digits were presented, gaussian white noise was presented at a level of 55 dBA in the six remaining speakers. The purpose of the noise was to facilitate an algorithm used to identify and suppress the electrical artifact produced by the CIs that contaminates EEG recordings (see below). Digits and white noise were processed in MATLAB 2009b (The MathWorks, Natick, MA, USA), and their presentation was controlled by a Tucker Davis Technologies (TDT, Alachua, FL, USA) RX8 Processor.

### Procedure

**Figure 1:**
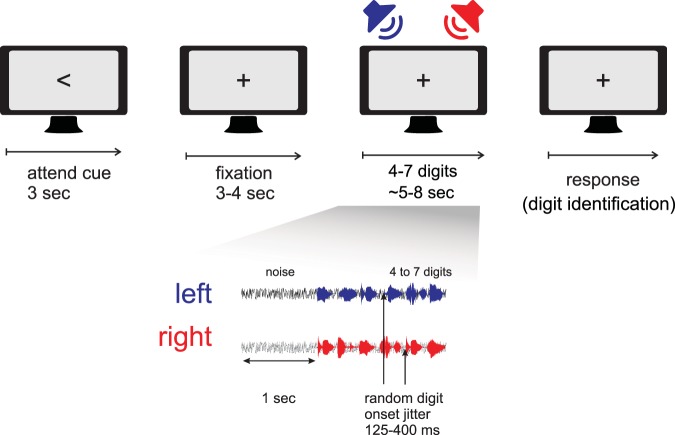
Schematic of experimental task. A visual cue prompted participants to either pay attention to the left or right speaker. Participants fixated on a cross on the computer monitor while two streams of digits were presented. Thereafter, participants reported the last digit that was heard on the attended side.

Detailed procedures of the task are schematized in Fig. 1. First, a visual cue appeared on a computer monitor positioned in front of the participant. The cue was either a left or a right arrow, indicating the speaker (left or right) to which the participant was required to attend, and lasted three seconds. The number of left and right direction cues was equal but randomized across the study. A fixation cross then appeared for a duration between three to four seconds, followed by the onset of gaussian white noise. After one second of white noise, digit stimuli were presented in both left and right speakers. Between four and seven digits were presented in the speakers on each trial. The number of digits on each trial was the same for either left- or right-sided presentations, and the number of digits on each trial was randomly and uniformly determined. Each digit was randomly chosen and could not repeat within the same speaker during the sequence, although the same digit could appear in the sequence between speakers. The offset-to-onset interval between digits was randomly and uniformly jittered from 125 to 400 ms. Thus, digit onsets between left and right speakers could occur simultaneously or shifted in time. The duration of digit and noise presentation was between five and eight seconds. White noise ended with the termination of the final digit. After, participants verbally reported the last digit they perceived on the side to which they were prompted to attend. The experimenter logged each verbal response.

The time period from the start of the cue to the response provided by the participant was considered one trial. Overall, trials were presented in eight blocks of 25 trials each, totaling 200 trials. Participants were able to rest for a short duration between blocks. The total duration of the study, from setup to completion, totaled three hours on average.

### EEG recording and preprocessing

The EEG was continuously sampled at 2000 Hz on a NeuroScan SynAmps II amplifier (Compumedics Ltd, Victoria, Australia). The EEG was recorded from 64 equidistant sensors on an ActiCAP (BrainProducts, Gilching, Germany) cap and referenced to a separate reference electrode positioned slightly anterior to the vertex. Positions of all electrodes and fiducials were digitized to a three-dimensional map using a Polhemus Patriot (Polhemus, Colchester, VT, USA). During recording, electrodes that overlapped or were next to the CI magnet and coil, between two and six electrodes across CI users, were not recorded.

Brain Vision Analyzer software (Brain Products, Gilching, Germany) was used to preprocess the data offline. First, a 2nd-order Butterworth filter was used to filter continuous EEG data from 0.1 to 40 Hz. Data were then downsampled to 250 Hz. Continuous EEG data were visually inspected, and trials in which there were large transient artifacts were marked. EEG data, excluding these marked epochs, were subjected to independent components analysis (ICA) to identify spatiotemporal patterns of biological artifacts that include eyeblinks, horizontal eye movement, and cardiographic activity. ICs were visually inspected, and those consistent with biological artifacts were set to zero before continuous EEG data were reconstructed. Across all participants, an average of 4.3 components were removed (SD = 0.99). Channels that were not recorded due to their proximity to the CI magnet and coil were then reconstructed using derived estimates from neighboring sensors using spline interpolation.

Preprocessed EEG data were then exported to MATLAB 2018a. Using the Fieldtrip toolbox^27^, continuous data were epoched from −1 to 10 seconds, relative to the onset of white noise at 0 ms. Trials with z-scored RMS amplitudes greater than 2 standard deviations away from the mean were removed. Between 0.5% and 17.5% of trials were removed across participants (M = 5%). After removal of trials containing artifacts, epochs were concatenated into a temporally continuous time series.

### CI artifact suppression in EEG

During auditory stimulation, CIs introduce electrical artifacts into EEG recordings. In order to suppress the CI artifacts, ICA methods have been widely used^28–30^. Here, we used a similar algorithm, second order blind identification (SOBI) implemented through the EEGLAB toolbox^31^, for minimization of the CI artifact. SOBI identifies independent components based on second-order statistics, making it suitable to separate temporally correlated signals^32^, such as the CI artifact. In addition, pilot studies we have completed suggest that the power-on artifact can be minimized during speech sound presentation by presenting it in low-level background noise at a level of 55 dBA, which was included in our stimulation design.

We importantly note here that ICA methods do not remove the artifact, but serve to suppress the presence of the artifact in the EEG recording. Further, the analysis and key inferences herein focus on *within*-subject differences of selective attention and how they relate to perceptual performance. As the CI artifact remains constant within each subject (i.e., between attend-left and attend-right conditions), its presence is thus controlled. CI artifact suppression is thus experimentally not needed in this design; however, we include it to facilitate data visualization.

SOBI was applied to continuous epochs of EEG data for each participant in the BiCI group. Topographical maps of IC weights were visually inspected, and time series of IC weights that matched the locations of the CI were plotted against the continuous sound stimuli that were presented for each participant. Component activations that mirrored the audio signal were set to 0 before reconstruction of the continuous epoched EEG. Between 0 and 2 components were removed per CI user. Finally, the EEG was re-referenced to the average of all EEG sensors before computing cortical tracking measurements and time-frequency analysis, which are described below.

### Cortical tracking of attended and ignored speech

To measure cortical tracking, we used a well-established linear forward modeling method to estimate the correspondence between the attended and ignored speech audio envelopes and continuous neural response in the EEG^33,34^. This technique uses ridge regression to estimate a linear filter that can be convolved with the speech signal to predict the EEG response. The filter is referred to as a temporal response function (TRF), characterized by weights across a range of time lags. Weights can be positive or negative, and like slope coefficients in regression models, describe how a change in the audio envelope magnitude corresponds to a change in the neural response magnitude. These changes are examined as a function of time lags, reflecting the delay between the time of change in the temporal envelope of the stimulus to the time of change in neural activity. For example, TRF weights at a time lag of +100 ms indicates the direction and degree of change of the EEG signal 100 ms after a change in the stimulus envelope.

Multivariate TRFs (or mTRFs) refer to modeling approaches that use multiple speech features (such as multiple speech streams, or representations of other acoustic features), whereafter each speech feature’s model can be assessed separately. Here, the attended and ignored speech stream were used as input features. We used the mTRF toolbox and procedures outlined by Crosse *et al*.^35^ in MATLAB to estimate TRFs over an integration window of −50 to 350 ms using ridge regression. We note here that the integration window is separate from the actual 11-second time epochs of EEG data. The integration window represents the range of time lags (or delays) between the epoch of EEG data and the stimulus envelope. Before TRF estimation, epoched EEG data were filtered from 1 to 8 Hz^36^. The speech envelope was calculated by taking the absolute value of the Hilbert transform for reconstructed speech signals. Time alignment of the speech signal to the EEG recording was conducted based on trigger events that were saved during EEG recording.

Ridge regression is associated with a regularization parameter that reduces overfitting the speech envelope to noise of the neural signal. *N-*fold cross-validation was used to optimize the regularization parameter across individuals^34^. During this procedure, TRFs are estimated for each individual for all but one of *N* trials. The resulting model is then used to predict the EEG signal during the trial that was originally omitted. Linear correlation (Pearson’s *r*) assessed the degree of agreement between the predicted EEG and actual EEG. The process is repeated for a range of regularization parameters from 0 to 8192 in powers of 2. Thereafter, these steps are repeated to predict all trials for each participant. Correlation coefficients were averaged across trials, channels, and participants. The highest correlation was for the regularization parameter of 2048, which was then used to estimate TRFs for each individual.

TRF magnitudes estimated for acoustic envelopes typically reach a maximum in the scalp topography at central and fronto-central sites in the EEG (e.g.^13^). Thus, for all TRFs, we selected channel Cz for analysis *a priori*. A second advantage of using this vertex channel was to avoid spurious responses that remained due to residual CI artifact, which arise most distinctly over temporal areas, and was confirmed by visual inspection.

TRFs were analyzed in a two-stage procedure. First, we tested for a difference between attended and ignored TRFs in each group separately by computing paired *t-*tests at each time lag. These tests were corrected for multiple comparisons by using nonparametric cluster-based permutation tests^37^ with 5000 randomizations. Due to the imprecision for temporal differences estimated by cluster-based permutation tests^38^, the outcome generally highlighted time regions for which more focused analysis could be conducted in a second stage. The second stage identified distinct peaks in the TRFs that could be extracted at an individual level. The magnitudes of these peaks were thereafter subjected to linear modeling for inference (see section on statistical analysis).

### Lateralization of alpha power

Time-frequency analysis was used to estimate alpha power during presentation of digit stimuli in order to examine alpha lateralization. Using the *Fieldtrip* toolbox, wavelet decomposition with 7 wave cycles was applied to single-trial epochs. Power was estimated across the frequency range of 2 to 30 Hz in 1 Hz intervals. Single-trial power values at each time point and frequency were then normalized by dividing power values by the average of the wide-band power during the whole epoch. After, single-trial power values were then averaged across the time range during which the digit stimuli were presented, since digit stimulation varied from trial to trial. For each individual, alpha power was taken at each individual’s peak value in the 7–14 Hz band after averaging across trials and sensors^39^. However, after peak alpha identification, these values were averaged separately for “attend left” and “attend right” conditions.

The convention for measuring alpha power lateralization involves computing a normalized “index” based on differences between the cued direction of spatial attention^16,40,41^. Two indices have been used. First, the Attentional Modulation Index (AMI; [*attend right* − *attend left*] */* [*attend right* + *attend left*]) indicates hemispheric changes in alpha power based on condition, with the expectation that positive values reflect larger alpha power in left hemisphere and negative values reflect larger alpha power in right hemisphere. Several studies have shown that the auditory alpha AMI contrasts most strongly in parietal sensors, and based on our equidistant EEG montage layout^42,43^, we selected two symmetrical parietal sensors that reflected the largest positive and negative AMI values pooled across both groups (see Fig. 3A).

The second index is Attentional Lateralization Index (ALI; [*ipsilateral* − *contralateral*] */* [*ipsilateral* + *contralateral*]), and summarizes the condition-based changes in alpha power, irrespective of left- and right-sided differences. The ALI is computed from the same sensors as the AMI, and is precited to be positive and larger if alpha power is higher on the side ipsilateral to the cued attentional direction. The ALI is the primary measure to compare neural suppression via alpha modulation between NH and BiCI groups.

### Statistical analysis

Statistical analyses were performed in MATLAB 2018a using the Statistics and Machine Learning Toolbox and in the R statistics package (R Core Team, 2019). Four groups of analyses were planned. First, a mixed analysis of variance (ANOVA) using the *lme4* package^44^ in R analyzed neural speech tracking. The model assigned group (BiCI vs. NH) as a between-subject factor, and within-subject factors were the attended versus. ignored stream, and peak response latencies that remained after permutation testing. For analysis of alpha power, since the metrics are normalized indices that remove individual differences in the overall magnitude, the AMI and ALI were tested using paired and unpaired *t-*tests, respectively.

The second set of analyses examined within-group interrelations between neural correlates of cortical speech tracking and the ALI. The objective of this analysis was to look at relationships that describe the balance of facilitatory and suppressive neural processes. For instance, comparison of neural tracking to the ALI compares how suppressive and neural facilitation relate during selective attention, and since different time lags represent different stages of the cortical auditory processing hierarchy^45^, the relationship between these different stages is of interest. Pearson product moment correlations were calculated between neural variables within each group. Correlations between groups were compared using Fisher’s *r*-to-*z* transformation.

In the third analysis group, linear regression was performed using the *fitlm()* function in MATLAB in order to predict performance on the speech streaming task from neural variables (cortical speech tracking, alpha lateralization) and the group. All two-way interactions that included the grouping factor were included in the model. Continuous predictor variables were *z-*scored prior to modeling. An ANOVA was used to assess the significance of predictor effects, and the effect size is reflected in the slope coefficient of each model term.

The alpha criterion for all tests was set to 0.05, and all tests were two-tailed. Test statistics are also reported alongside eta squared (*η²*) values to indicate effect sizes, with conventions of 0.01 as a small effect, 0.06 a medium effect, and 0.14 and above as large effects^46^.

## Results

### Early and late stage cortical selection of attended and ignored talkers

**Figure 2:**
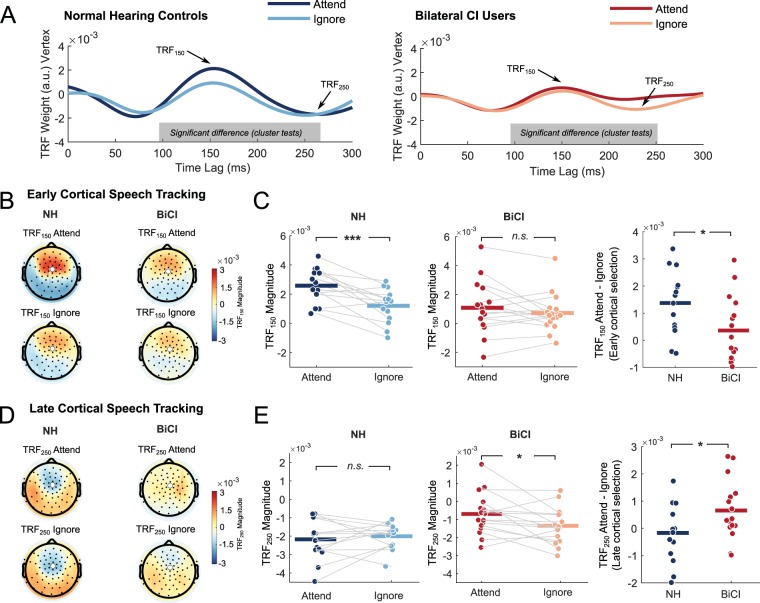
Temporal response functions. (**A**) TRFs at channel Cz for NH (left panel) and BiCI users (right panel). Grey shaded bars along the time axis represent the time regions that significantly differed between attended and ignored TRFs. Darker, saturated colors indicate the attended stream TRF, and lighter, less saturated colors indicate the ignored stream TRF. (**B**) Topographical maps of TRF weights at the ~150 ms time lag for attended and ignored stream for both groups. (**C**) In the left two panels, individual data points representing the peak TRF weight in the 100–200 ms time window for attended and ignored conditions for both groups. Lines connecting the dots show individual trajectories. In the rightmost panel, the difference between the attended and ignored conditions for the TRF_150_ weight, representing *early cortical selection*. (**D**) The same as B but for TRFs negatively peaking between 200 and 300 ms. (**E**) The same as C, but for individual-level TRF_250_ weights. The rightmost panel represents *late cortical selection*. *** = *p* < 0.001; * = *p* < 0.05; *n.s*. = not significant. All scalp images were created using *Fieldtrip* software and custom routines in MATLAB.

Cortical tracking of attended and ignored speech streams is shown in Fig. 2. Figure 2A shows the grand average TRFs for attended and ignored speech streams in the NH group (left panel) and BiCI group (right panel). As expected, the average TRF magnitude for the NH group was larger for the attended stream versus the ignored stream within the 100 to 200 ms time lag, and peaking near 150 ms. This difference was not as pronounced for the BiCI group. In contrast, cortical tracking during the 200 to 300 ms time lag was not descriptively large in the NH group, but was apparent in the BiCI group. In the latter group, cortical activity of the ignored talker was more strongly and negatively correlated to the speech signal than for the attended talker.

For NH individuals, results of the cluster-based permutation test comparing TRFs for attended and ignored speech returned one significant cluster from time lags of 96 ms to 264 ms (*p* < 0.001; Fig. 2A left panel). Similarly, BiCI users had one significant cluster spanning a comparable time range of 96 to 256 ms (*p* = 0.003; Fig. 2A, right panel). These time ranges appeared to cover two distinct peaks in the TRF, one at the aforementioned maximum at 150 ms, and another negative-going peak at 250 ms. Moving forward, these peaks are the focus of analysis. A peak-picking algorithm was implemented to identify the maximum TRF magnitude at this sensor in the time lag window between 100 and 200 ms. This range was chosen after visual inspection confirmed that this duration encompassed all peaks for all participants. Herein, this value is referred to as the TRF_150_ magnitude. In addition, the minimum value during the 200–300 ms time window was extracted for the attended and ignored stream for each individual. This value is referred to as the TRF_250_ magnitude. Note that the negative TRF peaks before the 100 ms time lag did not reach significance in the cluster-based test (consistent with Puvvada and Simon^47^), and thus are not considered further.

Figure 2B shows the topographical distribution of TRF_150_ weights averaged across each participant’s peak TRF magnitude at 150 ms. Whereas the NH group showed a strong difference in the TRF_150_ magnitude at frontal and central sensors, this difference was visibly weaker in the BiCI group. Individual data for each group and condition are plotted in Fig. 2C. The left and middle panel of Fig. 2C show that a majority of NH individuals had a larger TRF_150_ magnitude in the attend condition versus the ignored condition, whereas only about half of the BiCI users showed the same effect. This effect was reversed for the TRF_250_ magnitude in Fig. 2D,E; BiCI users appeared to have larger negative-going weights for the ignored stream, while NH individuals did not show a large difference in the attend and ignore streams near 250 ms.

One-sample *t-*tests, corrected for multiple comparisons using the procedure of Benjamini & Hochberg (1995), indicated that all TRF peaks were significant different from zero in both groups (all *p*s > 0.048). A 2×2×2 ANOVA indicated a main effect of group (*F*(1,28) = 4.49, *p* = 0.043, *η²* = 0.12), a main effect of attention (*F*(1,28) = 14.53, *p* < 0.001, *η²* = 0.05), and a significant group-by-latency interaction (*F*(1,28) = 13.23, p = 0.001, η² = 0.16). In addition, there was a significant three-way relationship between group, attention, and latency (*F*(1,28) = 9.44, *p* = 0.005, *η²* = 0.04). Since the three-way interaction implies that the main effects and two-way interactions are conditioned upon each other, the latter three-way interaction will only be considered further. In order to perform follow-up assessments, in each participant the difference between the attended and ignored streams was taken for the TRF_150_ and TRF_250_ magnitudes, reflecting the effect of attentional selection of the attended and ignored talkers during early and late stage cortical speech tracking. Herein, these differences are respectively referred to as *early* and *late cortical selection*. Early and late cortical selection values were compared between groups using two independent samples *t-*tests, which were corrected for their false discovery rate using the procedure of Benjamini and Hochberg^48^. Whereas the early cortical selection was larger for the attended stream in the NH group (unpaired *t*(28) = 2.31, corrected *p* = 0.046, *η²* = 0.16, see rightmost panel of Fig. 2C), late cortical selection was larger in the BiCI group (unpaired *t*(28) = −2.08, corrected *p* = 0.046, *η²* = 0.14, rightmost panel of Fig. 2E).

We also tested if the difference effects (Fig. 2C,E, rightmost panels) in each group were significantly different from zero, which would suggest that an effect was present even if it differed between groups. Early cortical selection was significantly different from zero in the NH group (paired *t*(13) = 4.32, p < 0.001, *η²* = 0.59) but not in BiCI users (p = 0.25). In contrast, late cortical selection was significantly different from zero in BiCI users (paired *t*(15) = 2.40, p = 0.030, *η²* = 0.27) but not in NH individuals (p = 0.56). The results imply that NH individuals and BiCI users differentiate attended and ignored speech at different cortical stages. Enhancement of the attended speech was observed near ~150 ms for NH controls, whereas the representation of ignored speech was stronger in BiCI users near ~250 ms.

### Spatial attention through the lateralization of alpha power

**Figure 3:**
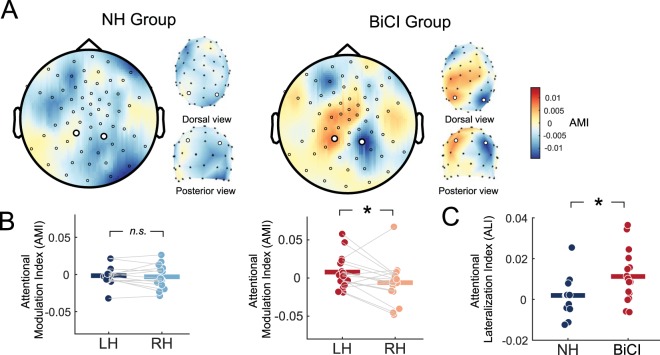
Ipsilateral suppression via alpha power modulation. (**A**) Topographies of the AMI for both groups. Large black circles represent sensors selected for analysis. Note that due to our equidistant layout, channel positions become distorted in 2D space near the vertex of the head. 3D plots of dorsal and posterior views adjacent to 2D topographies for each group shows accurate spatial locations of the sensors chosen for analysis. (**B**) AMI comparison for left hemisphere (LH) and right hemisphere (RH) sensors for both groups. Individual lines connect hemispheres within each subject. Thick colored bars represent the condition mean. (**C**) Comparison of ALI values between groups. Thick colored bars represent the group mean. * = *p* < 0.05.; *n.s*. = not significant. 2D and 3D scalp images were created using *Fieldtrip* software and custom routines in MATLAB.

Results of the alpha power lateralization analysis are depicted in Fig. 3. Topographical maps show the spatial distribution of the AMI during the time when digit stimuli were presented to NH individuals and BiCI users (Fig. 3A). The expectation was that positive AMI values would occur in parietal areas in the left hemisphere (larger alpha power during attend-left conditions) and negative values in the right hemisphere (larger alpha power during attend-right conditions), both reflecting suppression of the ignored speech. For NH individuals, the AMI between hemispheres did not appear to differ, whereas for BiCI users, the AMI in parietal sensors showed a stronger contrast.

Statistical results in Fig. 3B show that the AMI between left and right hemispheres was not significantly different in the NH group (*t*(13) = 0.55, *p* = 0.59, *η²* = 0.02), but was for the BiCI group (*t*(15) = 2.32, *p* = 0. 035, *η²* = 0.26). Comparison between groups using the ALI, and shown in Fig. 3C, revealed stronger alpha lateralization in the BiCI group compared to the NH group (*t*(28) = −2.23, *p* = 0.034, *η²* = 0.15). Overall, the results suggested that those in the BiCI group exhibited an effect of spatial attention through the modulation of alpha power, such that the hemisphere representing the ignored speech was associated with stronger inhibition. This was not observed for the NH group.

### Relationships between cortical tracking and spatial suppression

**Figure 4:**
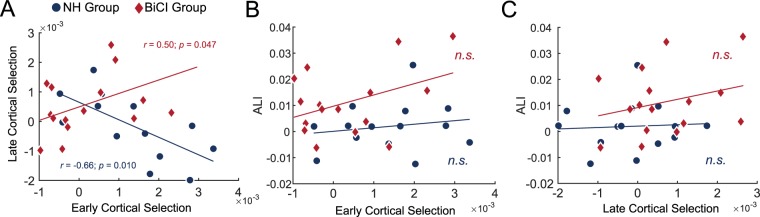
Interrelations between neural correlates of talker selection and spatial suppression for each group. (**A**) Relationship between early and late cortical selection. (**B**) Relationship between early cortical selection and alpha lateralization. (**C**) Relationship between late cortical selection and alpha lateralization. For all panels, lines represent the least-squares fit. *n.s*. = not significant.

Pearson correlations were used to examine relationships between early cortical selection, late cortical selection, and alpha lateralization. In the NH group, the early cortical selection negatively correlated to late cortical selection (*r* = −0.66, *p* = 0.010), such that the representation of the ignored talker was larger than the attended talker at the late cortical stage when this difference was small at the early cortical stage. The reverse was found for BiCI users. Early cortical selection positively correlated to late cortical selection (*r* = 0.50, *p* = 0.047). These relationships are plotted in Fig. 4A, and when compared using Fisher’s *r*-to-*z* transformation, were significantly different (*p* = 0.001). Alpha lateralization did not correlate to early cortical selection or late cortical selection in either group, and no group differences were found between group correlations (all *p*s > 0.12, Fig. 4B,C).

### Neural factors that predict task performance

**Figure 5:**
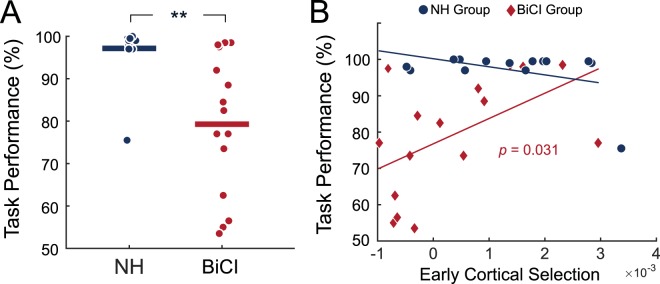
Group differences and predictors of task performance. (**A**) Difference in task performance between NH and BiCI groups. (**B**) Relationship between early cortical selection and task performance for each group. Lines represent the least-squares fit. ** = *p* < 0.01; *n.s*. = not significant.

Linear regression was used to predict task performance (reporting the final digit on the attended side) from early cortical selection, late cortical selection, alpha lateralization, and the experimental group (BiCI vs. NH). The regression model was able to capture nearly 40% of the variance in overall task performance (adjusted *R*^*2*^ = 0.398) and was significant when compared to a constant model (*F* = 3.73, *p* = 0.008). Examining main effects and interactions, a significant effect of experimental group (*β* = −15.88, *SE* = 5.70, *F*(1,22) = 9.77, *p* = 0.005) suggested that CI users had lower task performance compared to NH users (Fig. 5A). A significant interaction between experimental group and early cortical selection was found (*β* = 14.63, *SE* = 6.34, *F*(1,22) = 5.32, *p* = 0.031), and is plotted in Fig. 5B. The interaction suggested that early cortical selection significantly and positively predicted performance on the speech streaming task in BiCI users, but not for NH individuals. There was no evidence that late cortical selection or alpha lateralization related to performance.

## Discussion

### Summary

Here, we examined the neural correlates of multi-talker selective attention that differed between hearing-impaired CI users and normal-hearing listeners. The main findings are (1) cortical differentiation of attended and ignored talkers occurred at an early sensory processing stage (~150 ms) in NH listeners, while in BiCI users, differentiation occurred at a late cortical processing stage (~250 ms). (2) In NH listeners, stronger late cortical selection was observed when early cortical selection was weaker, but for CI users, early and late cortical selection were positively related. (3) BiCI users had stronger lateralization of alpha power in the hemisphere ipsilateral to the attended speech, suggesting suppression for the spatial location of ignored speech. This was not observed in the NH group. (4) Early cortical selection, but no other neural correlates, predicted BiCI users’ behavioral performance on the selective attention task. Overall, we conclude that neural evidence of selective attention is present in impaired listeners by way of late-stage cortical processing, as well as modulation of parietal alpha power, to potentially navigate a multi-talker situation. However, perceptual success appears to depend on how well attended and ignored talkers are differentiated at early stages of cortical processing.

### Early cortical tracking differences between impaired and unimpaired listeners

As found in our NH listeners, cortical entrainment is well known to be stronger for an attended talker over an ignored talker at a time lag near 150 ms^6,11,13^. Intracranial recordings have pinpointed this effect to nonprimary auditory cortical areas in superior temporal gyrus^5,12^, suggesting that object formation and talker selection begins in this region.

A weak cortical differentiation of the talkers with hearing impairment, as observed previously, suggests that cortical representations are not well distinguished during early cortical processing^24,25,49^. A likely explanation is that speech signals available to impaired listeners are lacking crucial spectral information that assist with spatial listening and perceptual object formation^21,25^. CIs for instance relay speech signals to the auditory nerve as modulated trains of electrical pulses, and while the envelope of speech (slower variations of amplitude over time) is transmitted, the temporal fine structure information (faster oscillations representing periodicities in the pitch content of speech) is severely diminished. Fine structure is similarly impoverished for impaired listeners^50^. Studies in NH listeners show that when the temporal fine structure of speech is removed, cortical speech tracking is less invariant to noise^36^ and the differentiation of talkers is significantly reduced^51,52^. Thus, temporal fine structure information appears to be essential when forming separate auditory objects during cortical processing at a latency of 150 ms, and our results reinforce this notion.

### Late cortical tracking differences between impaired and unimpaired listeners

A functional interpretation for neural speech tracking at late cortical stages (~250 ms) is presently unclear due to the lack of data under normal listening circumstances. More generally, recent research suggests that speech representations in the brain transform over time and in a hierarchy, where the earliest processing stages reflect properties of the acoustic signal, and evolve into higher-level linguistic units such as phonemes and words at later stages^45^. A difference between TRF magnitudes during late cortical selection was not statistically present in the NH group, presumably since differentiation of talkers was achieved at early cortical stages. Indeed, higher-level linguistic content is not a strong determinant of talker selection in NH individuals, whereas acoustic information is^45^. Interestingly, we found that NH listeners with larger late cortical selection had smaller early cortical selection. This “trade off” between early and late state cortical processing raises the tentative possibility that selection can shift to higher-level language information if early cortical selection is poorer.

BiCI users, on the other hand, had evidence of a stronger talker differentiation during later stages of cortical processing. A puzzle is that the representation for the ignored speech envelope was in fact stronger than for the attended speech envelope for this group, suggesting that attentional selection was not favoring the signal to which they were instructed to attend. Two alternatives that may explain this finding can be ruled out. First, this result cannot be attributed to a recently-reported late mechanism of cortical selection based on an active neural suppression of an ignored talker, since we did not find evidence that cortical tracking between talkers that was distinctly in the opposite polarity (i.e., positive vs. negative TRF weights) as has been a qualifier for ignored-talker suppression described previously^7,13^.

Second, BiCI users with stronger early cortical selection also had stronger late cortical selection (Fig. 4A). Thus, it does not appear that late cortical selection was compensating for poor early cortical selection, as was observed and suggested for NH individuals. Rather, the significant positive relationship could indicate that a strong differentiation of the talkers based on acoustics during early processing may have facilitated late selection, perhaps based on higher-level language features. Thus, it is incorrect to generally conclude that the ignored talker was, perhaps erroneously, overtly favored in BiCI users. For those BiCI users with generally stronger talker separation, a provisional interpretation is that selective attention may have unfolded in a two-stage process: The attended talker was first differentiated with a stronger representation at an early stage and perhaps based on acoustic differences between the talkers, and thereafter the ignored talker distinguished at a later cortical stage either based on acoustics or higher-level lexical features. Since this was markedly different from the early–late relationship for cortical selection in NH listeners, it is plausible that hierarchical stages of attentional selection to speech may be independent, or reliant on the degree of acoustic information available to the listener. We encourage further testing of this notion, however, owing to the ease of the task for the NH group.

### Suppression of ignored speech via spatial attention

BiCI users also exhibited larger alpha power that was lateralized to the hemisphere located ipsilateral to the attended talker, in agreement with the notion of neural suppression in the hemisphere where the ignored talker was represented^14–16,41^. This effect was absent in NH individuals. These modulations of alpha oscillations are believed to arise in supramodal (i.e., not specific to a sensory modality) regions of parietal cortex that are involved in the spatial focus of attention^17^. Notably, our configuration of results differed from those reported by Bonacci *et al*.^26^, who conversely found that impaired listeners had substantially weaker alpha lateralization compared to NH listeners. They concluded that impaired listeners were less able to use spatial cues to direct selective attention.

Notwithstanding the differences between the subject groups (here, CI users; Bonacci *et al*., unaided impaired listeners) and stimuli (here, speech; Bonacci *et al*., tone sequences), the disagreement in part could be explained by the degree to which talkers were perceptually distinguishable for the NH and BiCI groups. In a separate study, Bonacci *et al*.^41^ for instance demonstrated that alpha lateralization in spatial attention tasks was less pronounced when competing stimuli were perceptually distinct. Neural suppression related to spatial information processing was thus weaker when auditory objects were easier to form. NH listeners in our study appeared to perform at ceiling on the task, and speech entrainment for the attended talker was robustly differentiated from the ignored talker. For these individuals, it follows that spatial suppression of the ignored talker was likely not needed. Anecdotally, most of the NH listeners reported that the task was relatively easy and they could keep both left and right streams in memory.

This was not the case for the BiCI users, for whom the talkers were less perceptually distinct as evidenced in the task performance, would show an increase in spatial attention to compensate. Overall, the main conclusion surrounding the alpha lateralization results is that impaired listeners treated with a prosthesis, under the conditions of this study, were able to use spatial information to direct attention.

### Factors that explain difficulties with multi-talker perception in hearing impairment

BiCI users with a stronger differentiation between attended and ignored talkers during early cortical speech tracking were better at reporting the content spoken by the attended talker. This result again underscores the notion that early differentiation of speech (presumably based on acoustics^45^) is critical for perceptual selection and object formation^3,51,52^. Surprisingly, despite strong evidence for the presence of late cortical selection and stronger alpha lateralization in BiCI users, these effects were not predictive of task performance. If these selective attention neural correlates are behaviorally relevant for impaired listeners, they were not observed here. Above all, this finding may highlight why hearing-impaired listeners still have problems with selective attention in multi-talker scenes. Despite general neural evidence for multi-talker selective attention, perceptual ability may only be as successful as the strength of early cortical differentiation for attended and ignored talkers.

### Limitations and future directions

There are some notable limitations. First, no relationship was found between task performance and any neural variables in NH controls, likely because NH listeners found the task too easy and performed the task at ceiling. Thus, it is not possible to explain variability in their performance, and our conclusions are limited on how neural function underlying attentional strategies might be altered in impaired listeners compared to circumstances of normal hearing. In future studies, a more direct comparison to BiCI users may be afforded by presenting NH listeners with speech that has been vocoded to simulate listening through a CI^53–55^. These simulations may return a better performance match between NH and BiCI groups, and put a similar demand on NH listeners by limiting the degree of spectrotemporal information in the speech signal. Nonetheless, our results reflect the reality of how individuals with severe hearing loss face difficulty in simple multi-talker environments, and here we compared well-known neural correlates of selective auditory attention in this population for the first time.

Second, we used speech without semantic and syntactic information (i.e., natural speech) to better isolate effects of selective attention from linguistic influences. However, it is necessary to determine how our reported effects generalize to more ecologically valid listening contexts. Further to this point, a critical cue used in natural multi-talker listening contexts is visual information conveyed in movements of the talker’s mouth (i.e., lip reading) or in the talker’s gesticulation. Recently, concurrent visual information in multi-talker listening tasks has been shown to benefit attended-speech listening in normal-hearing individuals by inhibiting cortical responses to acoustic events designed to interrupt speech perception^56^, and visual information appears to improve neural tracking of speech-in-noise in individuals with age-related hearing loss^57^. Studies investigating the influence of visual information on cortical speech tracking of multiple talkers in hearing-impaired populations should be conducted to better understand underlying mechanisms selective attention.

Third, CI processors distort the acoustic envelope due to compression, filtering, and signal processing strategies^58^. Estimation of the neural response using the acoustic envelope may then be less optimal for the BiCI group. However, effects observed in this study were based on differences in within-subject comparisons, and because the task was counterbalanced between ears, issues with estimating TRFs affected each listener equally, and unlikely explain intra-subject attention effects.

A similar limitation concerns additional auditory processing challenges that impinge on between-ear cues that are available to CI users. For instance, due to variation in placement of the CI, there is a possibility of a frequency mismatch between ears such that the same pitch content is not equally delivered to both sides^59^. In addition, crucial timing cues that listeners normally use to localize sounds in space are poorly encoded^60^. The counterbalancing of attend-left and right conditions was designed to mitigate these individual-level factors of between-ear differences, but these challenges nonetheless contribute to the degradation of the acoustic signal and subsequent ability to encode these features. Thus, although the auditory processing challenges that face CI users in a multi-talker environment are more severe than expected for an impaired listener for whom a hearing aid would be beneficial, we demonstrate here evidence of neural processes that underly selective attention are still present in BiCI users and in part can predict their perceptual ability.

## Data Availability

The datasets generated during and/or analyzed during the current study are available from the corresponding author on reasonable request.
